# Flavivirus NS1: a multifaceted enigmatic viral protein

**DOI:** 10.1186/s12985-016-0590-7

**Published:** 2016-07-29

**Authors:** Meghana Rastogi, Nikhil Sharma, Sunit Kumar Singh

**Affiliations:** 1 Institute of Medical Sciences (IMS), Laboratory of Human Molecular Virology & Immunology, Molecular Biology Unit, Faculty of Medicine, Banaras Hindu University, Varanasi, 221005 India; 2 Laboratory of Neurovirology and Inflammation Biology, CSIR-Centre for Cellular and Molecular Biology (CCMB), Uppal Road, Hyderabad, 500007 India

**Keywords:** Flavivirus, Non-structural proteins, Arboviruses, NS1 protein

## Abstract

Flaviviruses are emerging arthropod-borne viruses representing an immense global health problem. The prominent viruses of this group include dengue virus, yellow fever virus, Japanese encephalitis virus, West Nile virus tick borne encephalitis virus and Zika Virus. These are endemic in many parts of the world. They are responsible for the illness ranging from mild flu like symptoms to severe hemorrhagic, neurologic and cognitive manifestations leading to death. NS1 is a highly conserved non-structural protein among flaviviruses, which exist in diverse forms. The intracellular dimer form of NS1 plays role in genome replication, whereas, the secreted hexamer plays role in immune evasion. The secreted NS1 has been identified as a potential diagnostic marker for early detection of the infections caused by flaviviruses. In addition to the diagnostic marker, the importance of NS1 has been reported in the development of therapeutics. NS1 based subunit vaccines are at various stages of development. The structural details and diverse functions of NS1 have been discussed in detail in this review.

## Background

Flaviviruses belong to the family of *flaviviridae*, which have 70 different antigenically related members. Most of the flaviviruses are arboviruses (arthropod-borne viruses). Arboviruses transmit mostly through ticks or mosquitoes bites. According to the outbreak reports, the Dengue virus (DENV), Japanese Encephalitis Virus (JEV), Yellow fever virus (YFV), West Nile virus (WNV) and tick-borne Encephalitis virus (TBEV) are prominent human-pathogenic flaviviruses. Recently, Zika virus outbreak has been reported in various countries and has become a matter of concern. Though, other flaviviruses (such as: St. Louis encephalitis virus, Murray valley encephalitis virus, Rocio virus, Kyasanur forest disease/Alkhurma virus, Omsk hemorrhagic fever virus and Powassan virus) are also pathogenic to humans but their geographical distribution is limited. The distribution and outbreaks of flaviviruses depend upon the geographic location of their vectors (mosquitoes and ticks) and reservoirs (birds and pigs) etc. [1].

**Fig. 1:**
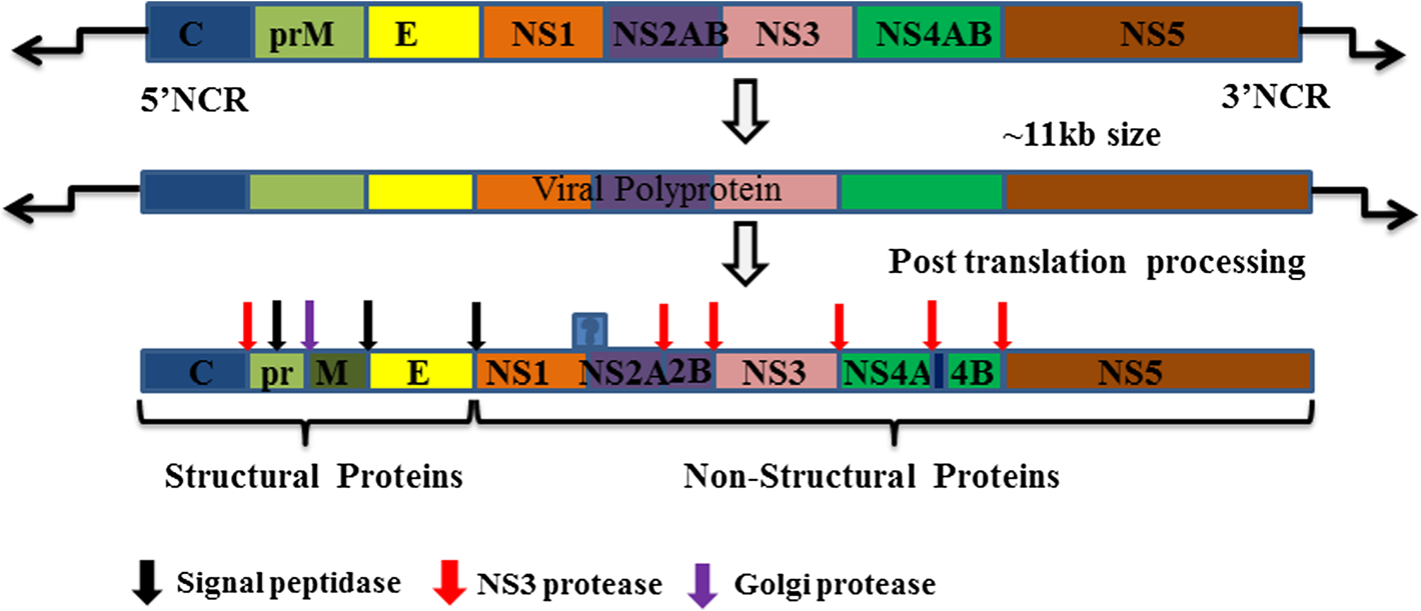
Flavivirus genome structure. The schematic diagram showing the processing of the flavivirus polyprotein into structural and non-structural proteins

Flaviviruses are enveloped, positive-sense, single-stranded RNA viruses with particle size up to ~50μm in diameter. The RNA genome of the flaviviruses contains the 5′ cap (7mG) and 3′ CU-OH conserved tail, which directly translates into a long polypeptide in the cytoplasm of infected cells. The polypeptide is further co-transnationally and post-transnationally cleaved and processed by host and viral proteases into three structural proteins: envelope protein (E), capsid protein (C) and precursor membrane protein (prM) and seven non-structural components (NS1, NS2A, NS2B, NS3, NS4A, NS4B and NS5) (Fig. 1). Among non-structural proteins, NS1 is highly conserved, dimer protein with the molecular weight ranges from 46–55 kDa depending on the extent of glycosylation. The glycosylation of NS1 is important for efficient secretion, virulence and viral replication [2–5]. NS1 exists as a monomer, a dimer (membrane-bound protein, mNS1) and a hexamer (secreted protein, sNS1). NS1 is known to activate the TLRs and inhibit the complement system [6, 7]. The intracellular NS1 is central to viral replication, whereas the secreted and membrane-bound NS1 have been reported to elicit the immune response [2, 8–10].

**Fig. 2:**
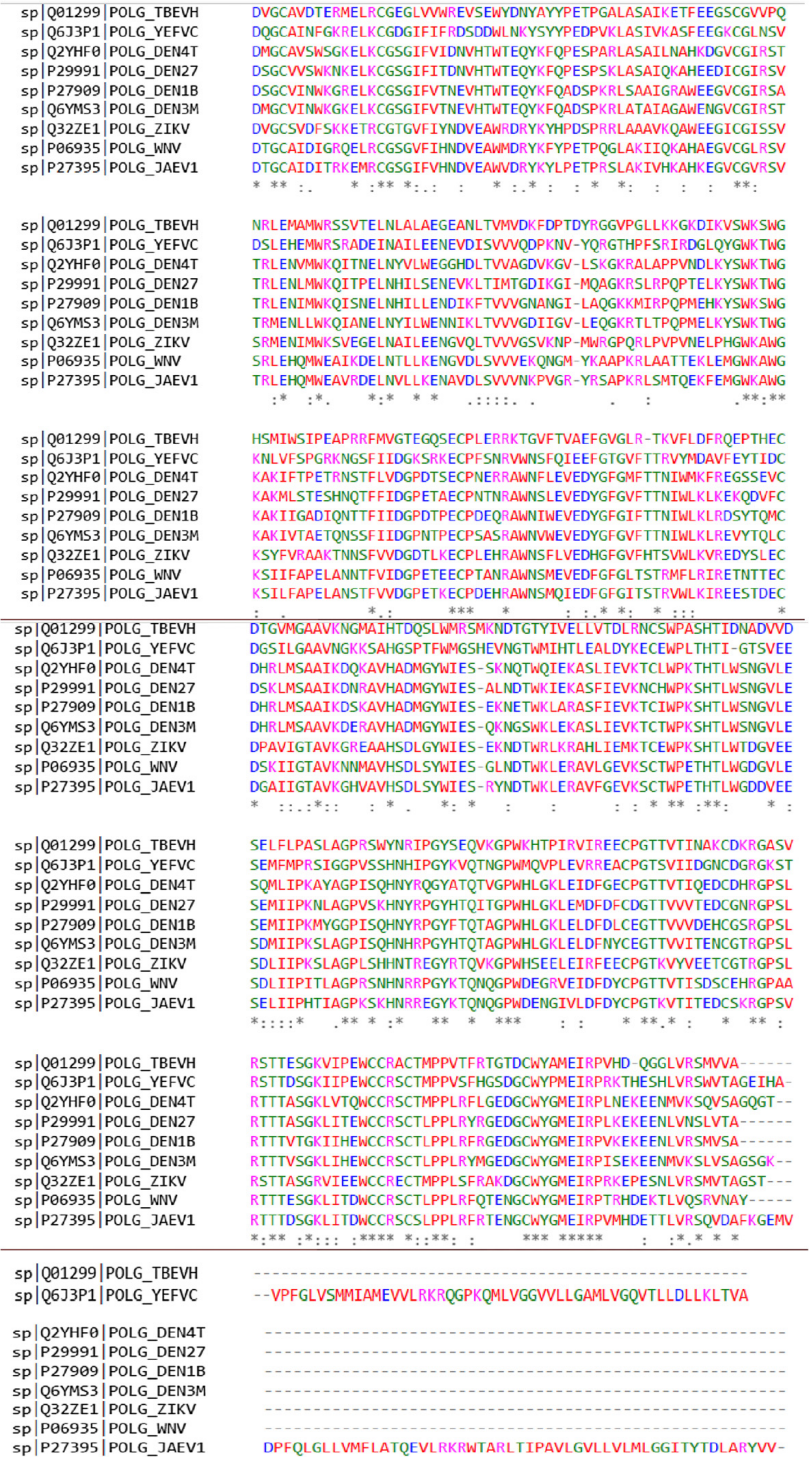
The comparative analysis of Flavivirus NS1. Comparative analysis of Flavivirus NS1 was performed by CLUSTAL Ω software, available on www.ebi.ac.uk/Tools/msa/clustalo/. The UniprotKB accession numbers for different flaviviruses are DENV-1 P27909, DENV-2 P29991, DENV-3 Q6YMS3, DENV-4 Q2YHF0, JEV P27395, TBEV Q01299, WNV P06935, YFV Q6J3P1, ZIKV Q32ZE1, and KUNJV P14335. The conserved sequences are shown by asterisk (٭), the amino acids that are strongly similar in their properties, are indicated by colon (:), those amino acids that are weakly similar, are represented by dot (.), while the dashes (−) represents the gaps among the sequences. The color-coding represents the different types of amino acids like, Red represents small and hydrophobic amino acid, including aromatic amino acid; Tyrosine. Blue represents acidic amino acids. Magenta represents basic amino acids. Green represents amino acids containing hydroxyl, sulfhydryl, amine groups, and Glycine amino acid. Grey represents unusual amino/imino acids

Comparative analysis of different flaviviruses NS1 has been performed by using a multiple alignment tool CLUSTAL Ω. This analysis provided the regions of similarity and dissimilarity among the NS1 sequences of different flaviviruses (Fig. 2). The CLUSTAL Ω based analysis revealed the conserved regions of NS1 among various flaviviruses (Fig. 2). The X-ray crystallographic 3-D structure of NS1 dimer suggests the three functionally distinguishable domains- a hydrophobic β-roll (a stretch of 1–29 amino acids), α/β Wing domain resembling RIG-I-like fold (38–151 amino acid residues) and a central β-ladder (181–352 amino acid residues), stabilized by disulfide linkages [11–13]. The association of NS1 with the membrane and replication complex is mediated through these three distinct structural domains. Yen et al. [14] reported the flexible nature of core β-ladder NS1 protein. JEV NS1 has been reported as a natural viral protein carrier for expressing the heterologous epitopes to stimulate the immune response against various pathogens [14].

The presence of sNS1 has been reported in circulation during primary and secondary infections and elicits higher concentration of IgG. The anti-NS1 antibodies have been reported to cross-react with broader range of host proteins like, human blood clotting factors, integrin/adhesion proteins; components of ECM [15]. The cell-adhesion assays revealed the interaction of fibronectin, plasma fibronectin and RGD (Arginine-Glycine-Aspartic acid) containing motif peptides with anti-NS1 (monoclonal and polyclonal) antibodies; which intervenes in the normal functioning of the vascular system. This probably forms the basis for the vascular leakage in DHF/DSS patients [16].

NS1′, an extension of NS1 protein has been reported in JEV, WNV and DENV [9]. The NS1′ is derived from viral polypeptide; through the cleavage due to the −1 programmed ribosomal frameshift slippage, downstream of NS2A protein. This results in the addition of extra 100 nucleotides to viral NS1 protein. NS1′ (52-53kDa) has been implicated in neuroinvasiveness of flaviviruses [17].

### General scheme of flavivirus replication

Flaviviruses are internalized into the cells by receptor-mediated endocytosis [18]. However, several putative receptors have been reported for the entry of flaviviruses, heparan sulphate proteoglycans, GRP78 (glucose-regulated protein 78), Hsps (Heat shock proteins) 70 and 90, CD14, CD4, cholesterol [19, 20]. Following their entry into the host cell, the acidic environment of endosome un-coats the nucleocapsid and releases its positive stranded RNA genome in the cytoplasm. The replication of flaviviruses takes place in two phases. In early phase of replication, the positive strand RNA behaves as mRNA and hijacks host factors for translation and cleavage of polypeptide in the ER lumen by host as well as viral proteases. In late phase of replication, the cleaved viral proteins interact with host cellular proteins [8, 21, 22]. The highly conserved secondary structures present in 5′ and 3′ UTRs of flavivirus ssRNA help in cyclization and synthesis of a complementary negative RNA strand; where the negative-intermediate copies serve as a template for the synthesis of new copies of the positive stranded RNA in membrane bound vesicles packets [23, 24]. The newly synthesized RNA copies from the nucleocapsid complexes bud into the ER lumen for obtaining the viral glycoproteins on the membrane. These immature particles then trafficked through the Golgi bodies and undergo maturation steps.

### Localization, maturation and secretion of NS1

The NS1 protein plays an important role in immune invasion and evasion strategies by modulating the host cellular machinery for effective virus propagation. Following the virus internalization the uncoating of nucleocapsid releases the viral RNA genome in the cytoplasm where the first round of replication occurs. The signal peptide in viral mRNA guides it to the ER lumen, where the membrane bound ribosomes translate them into structural and non-structural proteins. The polypeptide is cleaved by both hosts (signalases, furin) and viral serine proteases (NS3 serine protease). The C-terminus of E protein contains the signal peptide for cleaving and translocating the NS1 from translated polypeptide into ER lumen [25]. The C-terminus of NS1 have an octapeptide sequence (L/M-V-X-S-X-V-X-A), which is critical for cleavage and is found to be conserved in all flaviviruses. The production of antiviral therapies has targeted this octapeptide for the production of NS1-based vaccines [26]. The NS1/NS2A junction is also cleaved in ER lumen by some unknown proteases. The cleavage and translocation of NS1 is followed by the N-linked glycosylation in ER-lumen by glycosidase. Studies have revealed two glycosylation sites at Asn-130 and Asn-207, where higher orders of glycan and mannose carbohydrate moieties are added. The C-terminus of NS1 consist 6 pairs of cysteine residues which form disulphide bonds and are possibly involved in dimer formation, stabilizing the intracellular NS1 which imparts certain level of hydrophobicity. Although NS1 lack any membrane spanning domain, its association with plasma membrane is quite intriguing [11]. Akey et al. [11] and Watterson et al. [27] reported that the β-roll domain (10–11 and 159–162 residues) of NS1 dimer protrudes out and interacts with the ER lumen membrane. Akey et al. [12] have further reported the membrane rearrangement property of NS1 through an experiment, where they demonstrated the ability of WNV NS1 and DENV NS1 to rearrange the membranes of the liposomes [11, 12, 27]. The association of NS1 was reported to interact with cholesterol, lipid rafts and/or GPI anchor during dimerization [21, 28]. These findings suggest the interaction of intracellular NS1 with other viral protein and host cell during disease pathogenesis is necessary [11, 29].

The intracellular NS1 (dimer) along with other non-structural proteins and viral RNA are targeted towards ER, forming a replication complex (RC) from ER derived membrane structures called Vesicle Packets (VPs). RC plays an important role in active viral replication. In addition, the GPI-anchored NS1 and/or cholesterol/lipid raft bound NS1 gets trafficked to either plasma membrane through endocytic or secretory pathway or fuses with *trans*-Golgi network for further maturation and secretion [17]. The GPI-anchored form of sNS1 have been reported to affect the signal transduction pathways to modulate the pathogenesis of flaviviruses [30].

The trimming of carbohydrate moieties in cellular NS1 (dimeric form) takes place in *trans*-Golgi network by glycosidases and glycosyltransferases. These enzymes remove complex sugars from NS1 and form a soluble hexamer, which ultimately secretes out of the infected cell. Unlike mammalian cells used for protein expression, insect cells completely lack such process and accumulates NS1 inside the cell in in-vitro system [31]. The maturation in Golgi network is essential for NS1 secretion, since the trimming ensures the removal of mannose moiety and addition of highly complex sugars.

The sNS1 forms an open barrel shaped structure which is associated with 70 different types of lipid molecules. Further analysis of sNS1 revealed that the hexameric form have β-ladder and wing domain which provides an accessible area for interaction with hosts proteins, while the β-roll domain remains associated with central lipid channel [11].

### Role of NS1 in replication of flavivirus

Flaviviruses hijack host proteins in order to complete their genome replication and translation of viral polypeptide. Replication occurs in virus-induced membrane structure (IMS) derived from ER membrane known as Vesicle Packets (VPs) [32]. The remodeling ability has been shown by both NS4A, NS4B and NS1 proteins [12] which helps in formation of VPs [33].

The biochemical experiments in case of other flaviviruses have shown the association of NS1 with transmembrane NS4B [19, 29]. The transmembrane N-terminal of NS4A and NS4B interacts with hydrophobic β-roll of NS1 and stabilizes the transmembrane protein in the lumen of ER and helps in viral replication [29]. Muller et al. [17] have proposed a model where DENV RC is formed within VPs [17]. The Asn-130 plays role in viral replication while Asn-207 is involved in NS1 secretion from infected cells. The abrogation of glycosylation at Asn-130 in DENV serotypes 1 and 2 and YFV NS1 has been implicated in suppression of viral replication in both infected mammalian and insect cells [4, 34, 35]. However, Fan et al. [36] reported that the deletion mutant of Asn-207 in DENV-2 NS1, resulted into the suppression of viral replication in BHK-21 and *Vero* cells, which is inconsistent with the earlier reports [36].

### Functions of NS1′

NS1′ is an extended version of NS1 protein with the molecular weight of 52–53 kDa. The protein is identified in extracellular milieu during JEV, WNV and DENV infections [9, 37, 38]. Earlier it was speculated that NS1′ is produced by alternative splicing, downstream of NS2A gene while bioinformatics and mutational studies in WNV (Kunjin strain) identified a slippery heptanucleotide (YCCUUUU) followed by a pseudoknot structure at the N-terminal of NS2A gene [37]. The presence of both slippery heptanucleotide and pseudoknot structures result in −1 ribosomal frameshift; which leads to an addition of 52 amino acids at C terminal of NS1 genes [37, 39]. This additional peptide (FS52aa) was found to be immunogenic and raised antibodies in mice [40].

A single nucleotide mutation in JEV (G66A) and WNV (A30P) NS2A gene disrupts the formation of NS1′ leading to reduced neuroinvasiveness [37, 41] Satchidanandam et al. [42] and Takamatsu et al. [43] reported the association of flavivirus NS1′ with NS3 and NS5 inside replication complex in mammalian and avian cells [42, 43]. Later on, WNV NS1′ was shown to co-localize with NS1 in the ER and plays a role in viral replication where it substitutes NS1 function [44].

The secreted form of NS1′ has been reported in both infected mammalian and insect cells. The glycosylation events add higher order of mannose sugar to both sNS1 and sNS1′ in these cells. In mammalian infected and transfected cells, JEV NS1 and NS1′ secrete out slowly in extracellular milieu while in insect cells they retain back in cellular layers [9]. The retention of NS1′ was later on explained by the presence of 20 amino-acids at C-terminal of WNV NS1′ protein. The hydrophobic domains of NS1′ helps in attachment to the ER membrane and remain inside the cell [45].

### Interaction of NS1 with host proteins

Intracellular NS1 interacts with various host proteins to assist the viral replication, translation and virion production. The NS1 interacts with ribosomal proteins of 60S ribosome subunit: RPL18, RPL18a and RPL7. These ribosomal proteins are involved in translation as well as in some extra-ribosomal functions like, interaction with IRES (internal ribosomal entry sites) or anchoring the ribosomes to ER membrane. During flavivirus infection, NS1 interacts and re-localizes these proteins at the site of viral replication. siRNA based studies have shown the decreased viral translation, replication and virion production [46].

Heterogeneous nuclear Ribonucleoprotein C1/C2 and K [hnRNP C1/C2] are RNA binding proteins of nucleus and involved in mRNA processing, regulation of gene expression and maintenance of cellular homeostasis. During viral infection, hnRNPC and K proteins re-localize in cytoplasm and regulate the viral translation, replication, apoptosis in infected cell and disease pathogenesis [47, 48]. DENV NS1 along with hnRNP C1/C2, K and vimentin was reported to interact during DENV infection and help in virus propagation [49, 50].

Dechtawewat et al. [51] reported the co-localization of 36 cellular proteins with DENV NS1. Among them, NEK-2 (human NIMA-related kinase 2) regulates the cell-cycle; TOA-1 (thousand and one amino acid protein kinase 1) regulates apoptosis while COG-1 (component of oligomeric Golgi complex 1) has been reported in modification and transport of DENV NS1 [51].

Virus replication and translation process requires energy. Allonso et al. [52] reported the re-localization of GAPDH near viral replication by NS1 during DENV infection. The intracellular NS1 increases the glycolic flux, leading to glycolysis, which results in the release of energy utilized by DENV (16681 strain) during replication and translation [52].

The STAT family is involved in signal transduction and transcription factors upon activation by JAK kinases. STAT3β is an isoform of STAT3 α protein; member of STAT family. The SH2 and SH3 domains of STAT3 have been reported to interact with NS1 protein, which results into the release of TNF-α and IL-6 in response to the infection [53].

Identification of NS1 interacting partners will be helpful in elucidating the mechanism of pathogenesis of flavivirus infection.

### NS1 and Toll-like receptors

**Fig. 3:**
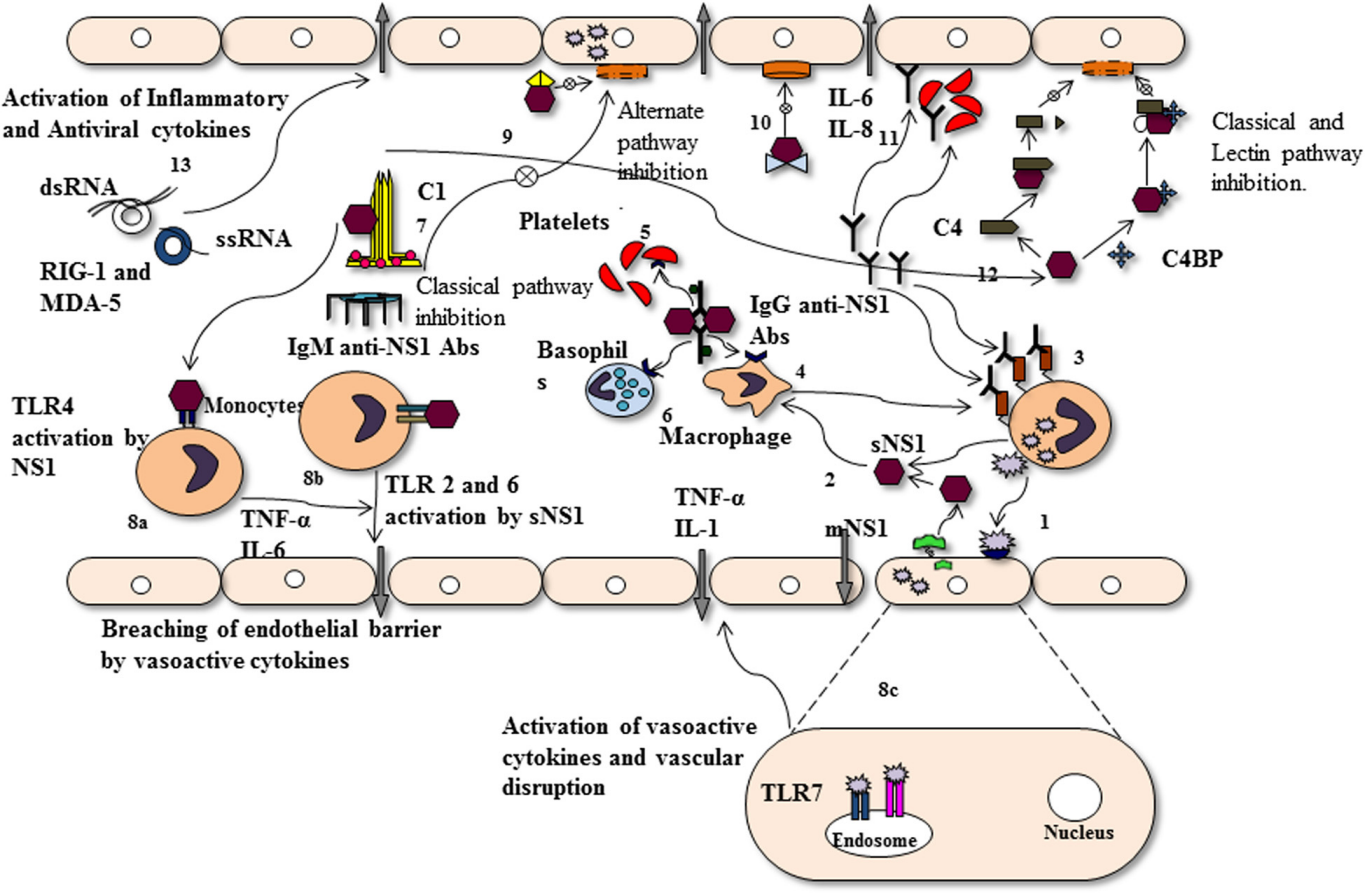
The diagrammatic representation of Flavivirus NS1 and its interacting partners. **1-**The infected cell releases the virion particles and infects the endothelial cells via several putative receptors present on cell surfaces, **2-3**-The secreted and membrane-bound NS1 interacts with several host immune cells like, Macrophages, Basophils, Monocytes and Platelets; along with IgG and IgM anti-NS1 Antibodies produce during Flaviviral infection, **4-6**-The immune complex (the anti-NS1 Abs and sNS1) activates the Fc-γ receptor present on those immune cells and release the vasoactive cytokines, which further increases the vascular permeability, **7**-The sNS1 efficiently interacts with IgM anti-NS1 antibodies and inhibits the classical pathway of complement system, **8**-The sNS1 activates the Toll-like receptors like2,3,4,6 and 7 present on cell surface or within endosomes of cell which further releases the inflammatory responses and disrupts vascular integrity, **9**-sNS1 associates with factor H of complement system and inhibits the membrane attack complex (MAC) formation, thus inhibiting the alternative pathway of complement system, **10**-The binding of sNS1 to clusterin protein activates the formation of C5b-9 and SC5b-9 formation which increases the cytokine storm, **11**-Also, anti-NS1 antibodies activate the platelet aggregation and/or Antibody-independent phagocytosis, which further aggravates the situation, **12**-The NS1-C1 interaction not only breakdown C4 into C4a and C4b but also interacts with C4BP, a recurring protein on infected cell and inhibits the aggregation of C3 and C4b on infected cell and prevent the MAC formation which ultimately blocks the classical and lectin pathway,**13**-The RIG-1 and MDA-5 helicases recognizes the single and double stranded RNAs and increases the activation of inflammatory and antiviral cytokines

The intracellular and sNS1 play a crucial role in both suppression and activation of various cellular responses during Flavivirus infection. RIG-I (retinoic-acid-inducible gene I), MDA-5 (melanoma-differentiation–associated gene *5*) and RIG-I-like receptors, (RLRs) recognize the viral RNA and respond via interferon signaling to protect the cell against viral infection (Fig. 3 (13)) [54]. TLRs are the membrane spanning PRRs (pattern recognition receptors), present either on cell surface or in endosomes and recognize PAMPs (pathogen associated molecular patterns) on invading pathogens. TLRs have been implicated in recognizing various flaviviruses, where they either increase or suppress the immune responses against pathogens.

There are contradictory reports on TLR3, where the presence of TLR3 augments WNV infection in mice model [55–57]. However, another study by Daffis et al. [58] reported inhibitory role of TLR3 during WNV infection [58]. Further, Baronti et al. [59] reported no role of NS1 in TLR3 signaling in WNV, YFV and DENV-2 [59], while Crook et al. [60] recently demonstrated the suppression of TLR3 signaling pathway by sNS1 based on in-vitro and in-vivo studies [60].

Activation of TLR2 and TLR6 by sNS1 during DENV infection has been reported to increase the production of proinflammatory cytokines like IL-6 and TNF-α (Fig. 3 8b) [61]. Modhiran et al. [62] reported the potential involvement of TLR4 in the recognition of sNS1 of DENV. The sNS1 has been reported to activate the macrophage and peripheral blood mononuclear cells (PBMCs) via TLR4, which led to the expression of proinflammatory cytokines. The increased production of these proinflammatory cytokines disrupts the integrity of endothelial cells. Furthermore, TLR4 antagonist and anti-TLR4 antibody treatment inhibited the vascular leakage, a characteristic hallmark of dengue disease severity. These findings suggest the interaction of NS1 with the TLRs [62].

The above mentioned reports show the dynamic role of intracellular and sNS1 during the Flavivirus infection. Also, how the NS1 is involved directly or indirectly during flavivirus pathogenesis.

### NS1 and complement activation

Complement system is an effector arm of antibody-mediated response against pathogens. It became evident through various studies that infected cells express both membrane-bound and sNS1 in extracellular milieu. Several proteins of complement system such as C3, C4, C5, clusterin, factor H (fH) and factor D have been reported to interact with sNS1 (Fig. 3). The sNS1 and membrane bound NS1 have been reported to activate the complement system via forming a membrane attack complex (C5b-9) and SC5b-9 (soluble terminal complement complex); which further increases the secretion of vasoactive cytokines resulting in disease severity, (Fig. 3) (9) [63]. Kurosu et al. [64] reported the interaction of sNS1 and clusterin, during DENV infection (Fig. 3) (10). They reported that clusterin inhibits the SC5b-9 formation but its interaction of sNS1 with clusterin increased the level of SC5b-9 [64]. The C4 protein has been reported to promote the inflammatory response. The sNS1 was found to stimulate classical/lectin pathway by binding and cleaving C4 to C4b; (Fig. 3) (12). Co-precipitation experiments revealed the interaction of sNS1 with proC1s/C1s and C4, this complex inhibits the concentration of C4 and protects the neutralization of infected cells. The extracellular sNS1 forms the complex with C4 and C4 binding protein1, which inhibits the viral recognition by host cell and protect the complement mediated lysis of infected cells in DENV, WNV and YFV [5, 65]. The plasma glycoprotein factor H (fH) is one of the crucial regulators of alternative complement pathway [66] and has been exploited by sNS1 in immune evasion strategy. The interaction of WNV NS1 with fH (WNV NS1-fH), degrades the C3b convertase and restricts the formation of C5b-9 membrane attack complex on infected cells [67]. However, Krishna et al. [68] did not find any interaction between JEV NS1 and fH complement protein [68].

The interaction of sNS1 with C1q has also been reported to play role in Antibody dependent enhancement (ADE) [69–71] (Fig. 3) (7–9). The crosstalk between sNS1 and acute phase proteins (APPs) leads to the enhanced acute phase response (APRs) in DENV infection. The APPs play important roles in innate immune response like, inflammation, infection, trauma or stress. The increased levels of APPs might lead to the pathophysiological conditions like thrombocytopenia, hemorrhage and disruption of vascular integrity as reported in DHF/DSS cases [72, 73].

### Role of NS1 in disease diagnosis and therapeutics

Quick adaptation of virus to changing environment, diverse geographical distribution and lack of effective vector control approaches ultimately led to the global burden of the flavivirus-associated diseases. Many strategies have been utilized for the diagnosis of flavivirus infections. The sNS1 protein in serum has been used as a diagnostic marker in flaviviral infections. Based on the clinical symptoms virus culture, RT-PCR and immuno-histological tools can be applied for detection of flavivirus infection [74]. The NS1 antigen-based ELISA has been used as a diagnostic tool in JEV, WNV and DENV infections [75, 76]. The newer approaches like the biosensor-based approaches, immuno-spot assays using fluorescent and opto-magnetic nanoparticles, electrochemical detection using carbon nanotube layering and surface plasmon resonance-based immuno-sensors have been tried at the various stages to quantify the NS1 from patient samples [77–80]. Several studies based on TLRs and NS1 interaction pointed out the use of TLR antagonists and anti-NS1 antibodies as therapeutics [61, 62, 81].

Various attempts have been made for NS1 based subunit and DNA vaccine against JEV, DENV and YFV. The NS1 based subunit vaccine (fusion protein of prM/E and NS1 protein or recombinant purified NS1) has been reported to be reactive against anti-NS1 antibodies and was found to be partially protective against infections [82–86]. Beatty et al. [81] reported the inhibitory effect of NS1-immune polyclonal serum and anti-NS1 mAbs on DENV-2 NS1 induced vascular leakage, both in vivo and in vitro [81]. To ascertain the safety and efficacy of NS1 based vaccine, the different formulations with different adjuvants were co-administered with recombinant NS1 in mice, which resulted into partial protection against flavivirus-induced lethal encephalitis [85, 87].

The NS1 based vaccine has been tested for its efficacy in flaviviral infections using the different constructs, DENV-NS1 lacking the C-terminal amino acids 271–352 (DCNS1), and chimeric DJNS1 (consisting of N-terminal DENV NS1 amino acids 1–270 plus) C-terminal of JEV NS1 amino acid 271–352. The modified forms of NS1 proved to be of better efficacy than unmodified NS1 in controlling flaviviral infections [84]. The NS1 based DNA vaccination against JEV and DENV have been reported to enhance the survival rate in mice by eliciting both cellular and humoral responses [88, 89].

Since NS1′ and NS1 co-exist in host cell, the expression of NS1′ revealed a negative effect on JEV infection and may have harmful effects on NS1 based DNA vaccines [90]. The cross-reactivity of WNV NS1 antibodies against JEV infection leads to a protective role; while DENV NS1 antibodies have a pathogenic role in increasing disease severity [15, 75, 90–94]. The purified recombinant NS1 protein either from the bacterial, yeast or mammalian expression system, retains its antigenicity and reported to be reactive against monoclonal or polyclonal anti-NS1 antibodies. Many in vitro and in vivo studies reported the partial or complete protection in response to polyclonal and monoclonal anti-NS1 antibodies treatment against mosquito-borne flaviviruses, reflecting the potentiality of NS1 as a vaccine candidate.

NS1 based vaccines are in various stages of development for providing better protection against the flaviviruses. Ishikawa et al. [86] developed a tripliVAX JE chimeric vaccine by substituting the JEV prM/E and WNV NS1 (RepliVAX JE) with JEV prM/E and JEV NS1 genes. The RepliVAX JE vaccine provided absolute protection from JEV infection by producing higher neutralizing antibodies in mice but its efficacy got compromised due to the presence of pre-existing anti-NS1 antibodies [86].

## Conclusion

Flavivirus infections have been reported as emerging infections over the past decades. The diagnostic and therapeutic tools are still a major challenge for most of the flavivirus infections but a significant progress has been made towards the development of such tools. The antibodies against the flaviviral NS1 proteins play a central role in prophylaxis and/or treatment of flavivirus infection through passive immunization. The higher concentration of NS1 directly correlates with disease severity and increased viremia. The NS1 protein is an important target for inhibitor design. The secreted and cell-surface-associated NS1 are highly immunogenic. The NS1 is an important biomarker for early diagnosis of the flaviviral infections. The information about the molecular structure and the interacting partners of NS1 provided better understanding about the role of NS1 in the pathophysiology of flavivirus-associated infections.

## Abbreviations

7mG, 7-methylguanosine; ADE, antibody dependent enhancement; APP, acute phase proteins; APRs, acute phase response; COG-1, component of oligomeric Golgi complex 1; DCNS1, dengue C’ terminal amino acid; DENV, dengue virus; DHF, dengue hemorrhagic fever; DJNS1, dengue N’ terminal amino acid; DSS, dengue shock syndrome; ECM, extracellular matrix; f(H), glycoprotein factor H; GAPDH, glyceraldehyde 3 phosphate dehydrogenase; GPI, glycophosphatidylinositol; GRP78, glucose-regulated protein 78; hnRNP, heterogeneous nuclear ribonucleo protein; Hsp, heat shock proteins; IMS, virus-induced membrane structures; IRES, internal ribosomal entry sites; JEV, Japanese encephalitis virus; MDA-5, melanoma differentiation associate gene-5; mNS1, membrane bound non-structural protein 1; NCRs, non-coding regions; NEK-2, human NIMA-related kinase 2; PAMPs, pathogen associated molecular patterns; PBMCs, peripheral blood mononuclear cells; PRRs, pattern recognition receptors; RC, replication complex); RGD, arginine-glycine-aspartic acid; RIG-1, retinoic acid inducible gene 1; RLRs, RIG-1 like receptors; RPL18/18a/7, ribosomal protein L18/18a/7; SC5b-9, soluble terminal complement complex; sNS1, secreted non-structural protein 1; STAT, signal transducer and activation of transcription; TBEV, tick-borne encephalitis virus; TLRs, toll like receptors; TOA-1, thousand and one amino acid protein kinase 1; UTRs, untranslated regions; VPs, vesicle packets; WNV, West-Nile virus; YFV, yellow fever virus
